# Report of Lecture on Ophthalmology and Otology

**Published:** 1881-11-20

**Authors:** 


### *REPORT OF LECTURE ON OPHTHALMOLOGY AND OTOLOGY.*

In this report we notice the following cheerful paragraph:

The practice, which at one time was so prevalent amongst physicians, as prescribing for any sort of inflammation of the eye an astringent collyrium, is happily far less common than it formerly was. Nevertheless, even at the present day, it is not without its adherents.

In reality, the field in which the use of astringents is called for, is comparatively circumscribed, especially since it has been of late encroached upon on the one hand by the adoption of boracic acid as a remedy in ophthalmic therapeutics, and on the other hand by the more extended use which is being made of the yellow oxide of mercury.

We clip the above from Lancet and Clinic, that we may give it our hearty indorsement. That eyes are often put out by astringent and irritating eye washes, we have no doubt, and this is not surprising when it is known that pure water alone, dropped into the healthy eyes every two or three hours, will in twenty-four hours, or less time, develop an acute ophthalmia.

In the purulent ophthalmia of infants we have long since abandoned the use of eye waters. Frequent bathing the eyes externally with tepid water, and anointing the edges of the lids with vaseline, we have found a safe and efficient treatment. The vaseline is designed to prevent the agglutination of the lids by the encrusted pus and its accumulation upon the ball.

